# Recent Advances in Boron-Containing Conjugated Porous Polymers

**DOI:** 10.3390/polym8050191

**Published:** 2016-05-12

**Authors:** Feng Qiu, Wuxue Zhao, Sheng Han, Xiaodong Zhuang, Hualin Lin, Fan Zhang

**Affiliations:** 1 School of Chemical and Environmental Engineering, Center of Graphene Research, Shanghai Institute of Technology, Shanghai 201418, China; fengqiu@sit.edu.cn (F.Q.); hansheng654321@sina.com (S.H.); 2 School of Chemistry and Chemical Engineering, State Key Laboratory of Metal Matrix Composites, Shanghai Jiao Tong University, Shanghai 200240, China; zhaowuxue@yeah.net (W.Z.); zhuang@sjtu.edu.cn (X.Z.)

**Keywords:** conjugated porous polymer, boron chemistry, structural characteristics, physical properties, application

## Abstract

Porous polymers, integrating the advantages of porous materials and conventional polymers, have been well developed and exhibited tremendous attention in the fields of material, chemistry and biology. Of these, boron-containing conjugated porous polymers, featuring tunable geometric structures, unique Lewis acid boron centers and very rich physical properties, such as high specific surface, chargeable scaffold, strong photoluminescence and intramolecular charge transfer, have emerged as one of the most promising functional materials for optoelectronics, catalysis and sensing, *etc.* Furthermore, upon thermal treatment, some of them can be effectively converted to boron-doped porous carbon materials with good electrochemical performance in energy storage and conversion, extensively enlarging the applicable scope of such kinds of polymers. In this review, the synthetic approaches, structure analyses and various applications of the boron-containing conjugated porous polymers reported very recently are summarized.

## 1. Introduction

Over the past few decades, porous materials, like activated carbons [1,2,3], zeolites [4,5] and metal organic frameworks (MOFs) [6,7,8], with high surface areas and well-defined porosity have been well developed and widely used in many fields. Different from inorganic porous materials, porous polymers [9,10,11,12,13], featuring micro-/mesoporous scaffolds with the advantages of controlled porosity, tailorable chemical compositions, multiple functionalities and convenient processibilities, have received much attention in gas storage and separation [14,15,16,17,18,19], catalyst [20,21,22], proton exchange membrane [23], sensor [24,25,26,27,28], drug delivery [29,30,31], biomolecular immobilization and tissue scaffold [32,33,34,35], ultrahigh electrochemical capacity [36,37,38,39,40,41], *etc.* According to their structural architectures, porous polymers can be classified as hyper-crosslinked polymers (HCPs) [42,43], polymers of intrinsic microporosity (PIMs) [44,45], conjugated microporous polymers (CMPs) [36,46], covalent organic frameworks (COFs) [10,47], porous aromatic frameworks (PAFs) [48,49,50], *etc.*, which are efficiently achieved via various chemical reactions, including anionic polymerization [51], addition-fragmentation chain transfer polymerization (RAFT), polycondensation [10,11,52,53,54], transition metal-catalyzed cross-coupling reactions [55,56,57,58,59] and click reactions [60,61,62]. With the help of these efficient synthetic protocols, incorporating some key components into the polymeric scaffold plays an important role in giving rise to porous polymers with promising properties.

Post-synthesis and the bottom-up approach have been considered as the two main strategies for the functionalization of porous polymers. For the former case [63,64,65], the building blocks in a porous polymer network can be effectively modified, which is then linked with the functional moieties by chemical transformation or coordinated binding. In the case of the latter one [39,66,67,68], functional moieties are directly introduced into the building units of a porous polymer prior to synthesis. For instance, a porous β-cyclodextrin polymer formed by polymerization of β-cyclodextrin tetrafluoroterephthalonitrile showed high surface-area and a mesoporous architecture, which could rapidly remove the organic micropollutants from water through the host-guest interaction [68]. Recently, heteroatoms were found to serve as efficient elements for constructing functional porous polymers [36,38,69]. For example, some porous polymers combining electron-withdrawing heteroatom moieties, e.g., boron atom or benzothiadiazole units, with *p*-type building blocks, e.g., triethynylbenzene, tetraphenylethene or triphenylamine, would generate charge/energy transfer between donor/acceptor components, significantly expanding the applications of porous polymers in light-emitting devices, metal-free catalysts and advanced electrodes [46,57,69].

To incorporate heteroatoms into porous polymers, early work was focused on the post-synthetic method. For example, porous polymers were activated by the reaction of ammonia (NH_3_) with carbon of the network at high temperatures [70]. However, this approach would destroy the skeletons of porous polymers in the process of the formation of nitrogen-doped carbon materials. Alternatively, the bottom-up methods on the basis of the different performances, including transition metal-catalyzed cross-coupling polymerization [36,46], solvothermal polycondensation [71] and electrochemical polymerization [72], have been regarded as the convenient approaches for the exact construction of heteroatom-containing porous polymers. In this regard, heteroatoms can be incorporated into the frameworks of porous polymers through the polymerization of heteroaromatic-based building blocks, like melamine [38,73], cyanobenzene derivatives [74,75,76], thiophene derivatives [77,78,79], triphenylphosphine [80,81,82,83], triarylboranes [28,57,58,84], *etc.* On the other hand, the formation of heteroatom-containing linkers (e.g., imine [38,53,73], acylhydrazone [54], diazaborine [85], thiol–yne [86]) via the polycondensation of the building blocks can be taken into account to couple heteroatoms into the networks of porous polymers. The species, quantities, pore size distribution, as well as valence states of heteroatoms in the porous polymers can be well controlled by such a bottom-up performance, thus allowing one to clearly understand the relationship between structure and properties.

To date, many heteroatoms, such as nitrogen, sulfur, phosphorus and boron, have been coupled into the skeletons of porous polymers. Among these light elements, boron, a IIIA element in the periodic table, is an indispensable one in organic chemistry [87,88,89]. Due to a boron atom possessing three valence electrons, tri-coordinate boron structures can be formed by sp^2^–hybridization. Furthermore, a four-coordinated boron-cored unit would be obtained by the coordination of the empty p orbital on each boron center with a lone pair of electrons of a heteroatom or anion ion [89,90]. Encouraged by its alterable chemical bonding, the boron atom enables enriching the functions of organic materials, including reagents, catalysts and luminescent materials [87,90,91]. With the continuous emergence of new organoboron dyes and polymers with fantastic properties [88], the boron-containing porous polymers have attracted increasing interest in the applications of luminescent devices, catalysts, energy storage and conversions. Previous reviews concerning boron-containing porous polymers mainly reported the boron-linked COFs [13,47]. In these systems, boron-carbon linkages belong to dynamic covalent bonding, leading to forming of heteroatom-containing porous polymers with high crystallinity. However, some intrinsic characteristics of the boron atom, for example Lewis acidity widely known in tri-coordinate borane-containing organic small molecules and polymers [20,21,22], are still not explored in porous polymers until recent years.

**Figure 1:**
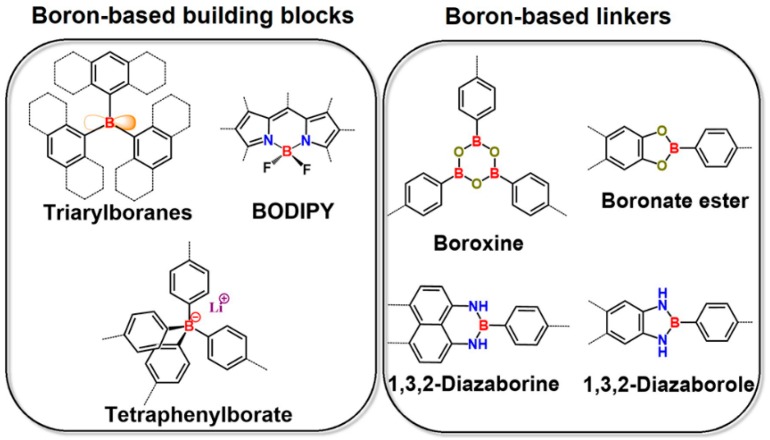
The categories of boron-based building blocks (left) and boron-based linkers (right) used for boron-containing conjugated porous polymers.

Normally, the boron atom is employed as a key component in a building block or linker for the formation of a boron-containing conjugated porous polymer network (Figure 1). In the former case, several kinds of boron-based functional molecules, including triarylboranes [28,57,58,84], boron-dipyrromethene (BODIPY) derivatives [46,92] and tetraphenylborate [93,94,95,96], have been used as building blocks for the preparation of porous polymers through, for example, carbon–carbon cross-coupling reactions, and the boron atom normally exerts a crucial effect on either the physical properties or the main functions of a resulting porous polymer. For the latter case, the boron-based heterocyclic linkers consisting of multiple B–O or B–N covalent bonds [47,85], which are formed via the condensation reaction, represent one of the most popular dynamic covalent bonds for the construction of a high crystalline framework (COF), and the boron atom mainly serves as a linkage for the formation of a network, but has less effect on the functionality of a target porous polymer. According to these remarkable differences between these two categories above, the representative examples in the recent reports are presented in the following sections.

## 2. Networks with Different Boron-Based Building Blocks

### 2.1. Triarylborane-Based Building Blocks

In the past several years, various tri-coordinate boron compounds, such as diboraanthracene, borafluorene, anthrylboranes and triarylboranes, have been reported. Among them, triarylborane is one of the most important series of organoboron compounds [87,97]. Owing to the steric protection provided by the bulky aryl moieties, many triarylboranes exhibit excellent air and moisture stabilities. Furthermore, the sp^2^–hybridized boron center, isoelectronic with carbocation, overlapping its empty p_π_-orbital with the adjacent organic π-conjugated system (e.g., aryl, vinyl, alkynyl) in a boron-based chromophore, would lead to the formation of an extended π-conjugated backbone with a strong electron-deficient character, with respect to the rich photophysical properties of such kinds of molecules. Therefore, the boron-based chromophores have become one of the key active components in many electronic devices, such as nonlinear optical emitters and electroluminescence diodes [87]. Therefore, the construction of various polymeric materials on the basis of triarylborane-based building blocks from linear conjugated polymers to porous polymers by the copolymerization with other monomers is highly desirable.

**Figure 2:**
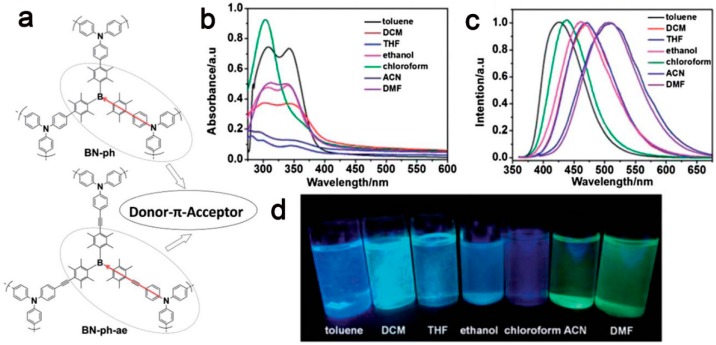
A structural illustration of **BN-ph** and **BN-ph-ae** embedding D-π-A units (**a**); normalized UV-VIS absorption spectra (**b**) and fluorescence spectra (**c**) (λ_ex_ = 350 nm) of **BN-ph**; luminescence photographs of **BN-ph** in different solvents under UV irradiation at λ_ex_ = 365 nm (**d**). Reprinted with permission from [57]. Copyright 2013, The Royal Society of Chemistry.

In 2013, our group reported two kinds of multi-functional conjugated porous polymers (**BN-ph** and **BN-ph-ae**) bearing trisdurylborane and triphenylamine as building block via Suzuki cross-coupling polymerization and Sonogashira cross-coupling polymerization, respectively [57] (Figure 2). They exhibited amorphous features with a classic disordered porous structure. The porous properties of **BN-ph** and **BN-ph-ae** were investigated by nitrogen sorption analyses, which indicated that both of them exhibit Type II isotherms. The high specific surface area (*S*_BET_) of 1279 m^2^·g**^−^**^1^ were achieved for **BN-ph**. We found that *S*_BET_ of **BN-ph-ae** dropped to 634 m^2^·g**^−^**^1^, with the increasing of π-conjugated length in the porous polymer. The reason was probably attributed to the longer spacer employed by acetylene-mediated **BN-ph-ae** than that of **BN-ph**. Moreover, **BN-ph** and **BNph-ae** showed H_2_ uptakes of 143 cm^3^·g**^−^**^1^ (1.28 wt %) and 106 cm^3^·g**^−^**^1^ (0.95 wt %), respectively, at 1 bar and 77 K, which was in good agreement with their surface area results. Owing to the existence of the D-π-A unit in **BN-ph**, the nearly solvent polarity-independent UV–VIS absorption was obtained, indicating a relatively small dipole moment in the ground state; while the emission maxima of **BN-ph** was red-shift from 427 to 509 nm with the increasing polarity of solvents. This suggested a substantial polarized structure of **BN-ph** in the excited state, which was in accordance with a typical D-π-A-conjugated system [98,99,100]. However, the **BN-ph-ae** with a longer π-bridge between D and A showed a weak influence on this solvatochromic behavior, indicating the efficient D-π-A conjugated system with the push-pull effect in the whole frameworks. Upon excitation at 350 nm, **BN-ph** with a shorter π-bridge had an intense luminescence with a quantum yield (Φ) of 0.13; **BN-ph-ae** showed much weaker emission with Φ of 0.07, which was consistent with the results of the photoluminescence lifetime. Such a difference could be attributed to the efficiency of the charge transfer emission through the π-bridge [101]. Both **BN-ph** and **BN-ph-ae** also exhibited good solid-state photoluminescence performances, which might be beneficial from the rigid skeleton and amorphous architecture, thus leading to the restriction of nonradiative deactivation and the π-π interaction in the solid state. On the basis of their excellent luminescent properties, **BN-ph** could be used as probe for the detection of fluoride. With the increasing of the molar ratio of n-Bu_4_NF (TBAF) in THF dispersion, the emission intensity of **BN-ph** gradually decreased, caused by the suppression of charge transfer from nitrogen to the boron center through the coordination of fluoride anions with boron atoms.

In the same year, Liu and coworkers reported the preparation of two kinds of triarylborane-based conjugated microporous polymers (**BCMP**) in high yields [58]. One was prepared via an alkyne-alkyne homocoupling of tris(alkynylduryl)borane (**BCMP-1**); another was synthesized through Sonogashira–Hagihara coupling copolycondensation of tris(iododuryl)borane and tri(4-ethynylphenyl)amine (**BCMP-2**). Surface area values of 815 and 911 m^2^·g**^−^**^1^ were obtained for **BCMP-1** and **BCMP-2**, respectively, which are much higher than that of the full-carbon acetylene-mediated CMP network. Owing to the good stability and excellent porosity characteristics of BCMPs, the CO_2_ adsorption capacity of these BCMPs was determined. **BCMP-1** could store up to 55.1 mg·g**^−^**^1^ of CO_2_ at a temperature of 273 K and 1 bar pressure, which are comparable with the reported boron-based COFs with higher surface area and larger pore volume; while a high carbon dioxide uptake of 74.5 mg·g**^−^**^1^ was obtained by **BCMP-2** containing triphenylamine under the same conditions, demonstrating that the electron-rich nitrogen atoms on the pore wall of **BCMP-2** have a positive influence on the uptake capacity of carbon dioxide through the strong acid-base interaction [102]. Besides, these BCPMs also showed good photophysical properties. The UV–VIS absorption spectra of **BCMP-1** synthesized from homocoupling of tris(alkynylduryl)borane is red-shifted from that of its monomer by 58 nm, indicative of the extended π-conjugation for the porous polymer. Upon irradiation from a UV lamp, strong luminescence with emission maxima (λ_em_) at 483 nm was observed. For **BCMP-2**, the UV–VIS and photoluminescence (PL) spectra are remarkably red-shifted by 30 nm by comparison to those of **BCMP-1**, suggesting the electron-donating effect of nitrogen in triphenylamine. The emission behavior of **BCMP-2** is strongly dependent on the polarity of organic solvent, due to the intramolecular charge transfer (ICT) from donor (D) nitrogen to acceptor (A) boron. In 2015, the same group reported a boron-containing conjugated porous polymer **BCMP-3** with high surface area (950 m^2^·g**^−^**^1^) by Suzuki cross-coupling polycondensation of tris(4-bromo-2,6-dimethylphenyl)borane and tris(4 dihydroxyboranylphenyl)amine [84]. This polymer was explored as a porous luminescent chemosensor for selectively detecting trace amounts of F**^−^** over other common anions. Moreover, **BCMP-3** could work as an adsorbent for F^−^ removal and showed good adsorption capacities of up to 24 mg·g^−1^ at equilibrium F^−^ concentrations of 16 mgL^−1^ and a temperature of 298 K. Owing to the low solubility in organic solvent, it exhibited good recyclability and reusability for F^−^ removal without the loss of adsorption efficiency.

**Figure 3:**
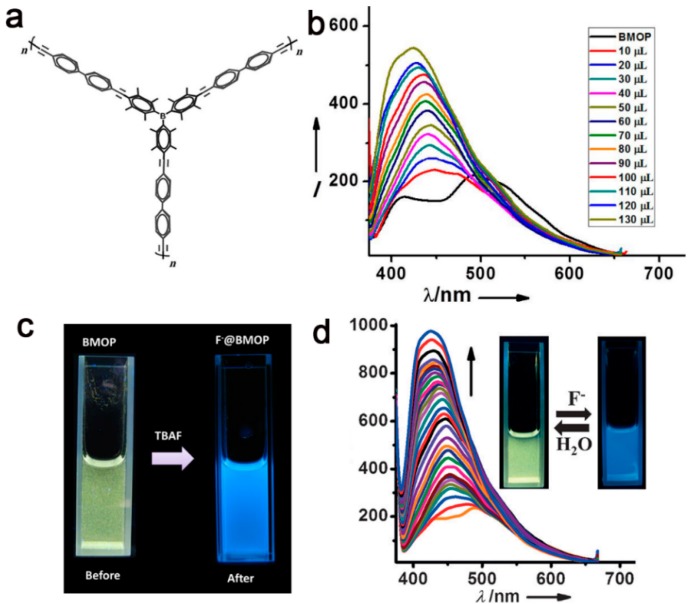
A structural illustration of **BMOP** (**a**); fluorescence spectra of **BMOP** on incremental addition of F- ion in THF solution (**b**); luminescence photographs of **BMOP** under a UV lamp before and after the addition of TBAF in THF (**c**); TBAF concentration-dependent fluorescence spectra of the **BMOP** THF/H_2_O (9:1) mixture; inset: digital images of **BMOP** THF/H_2_O mixture with the addition of F**^−^** under 365-nm UV light (**d**). Reprinted with permission from [28]. Copyright 2015, Wiley-VCH.

Similarly, Maji *et al.* [28] developed a boron-containing porous polymer (**BMOP**) consisting of 2,3,5,6-(tetramethylphenyl)boron and diethynylbiphenyl, which showed the formation of clustered spherical particles with a dimension of 100 to 300 nm. A *S*_BET_ of 390 m^2^·g^−1^ with an average pore size of 1.08 nm was obtained (Figure 3). Upon the treatment with fluoride anion, **BMOP** showed ratiometric fluorescent properties, in which the intensity of the emission band at 520 nm decreased, while the emission band at 420 nm was enhanced. Accordingly, the fluorescent color of **BMOP** solution changed from green to blue. Such a phenomenon could be attributed to the block of ICT from donor to acceptor, resulting in π-π* emission localized on the extended monomer of the tetramethylphenyl-fused 1,4-diethynylbiphenyl unit. Particularly, the detection of F^−^ in water would be carried out in a mixed solvent of THF/H_2_O. This ratiometric fluorescent change also could be observed distinctly, and the detection limit of F^−^ ion by **BMOP** could reach to 2.6 μM, due to the strong B–F interactions. Upon the addition of an excess of water, F^−^ in **BMOP** was eluted, and correspondingly, green emission was recovered. Therefore, the real-time monitoring of the selective capture or release of F^−^ ions would be realized.

### 2.2. Triphenyl Borate-Based Building Blocks

**Figure 4:**
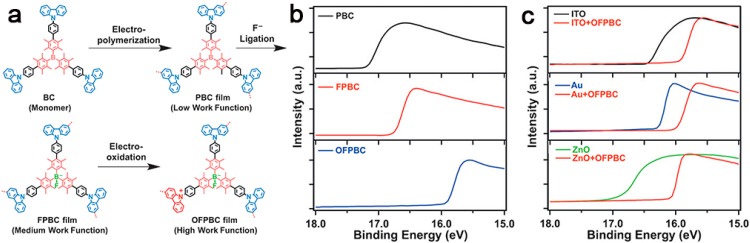
A synthesis route of ionic **OFPBC** films (**a**); secondary electron cut-off obtained by ultraviolet photoelectron spectroscopy for **PBC**, **FPBC**, and **OFPBC** films (**b**); ITO, Au and ZnO samples with and without 20 nm-thick **OFPBC** films. The shift represents the change of the work function (**c**). Reprinted with permission from [72]. Copyright 2016, Wiley-VCH.

Each Lewis acid boron center in a tri-coordinate boron-based porous polymer allows for accepting a lone pair of electrons from some anions, like fluoride [90] and cyanide [103,104], to form an anionic tetra-coordinate boron-based porous polymer network. In this context, Jiang and coworkers [72] recently synthesized a key monomer bearing a tris(2,3,5,6-tetramethylphenyl) borane core with three N-substituted carbazole groups at the periphery. Then, the triphenyl borane-based porous polymers with a surface area of 1074 m^2^·g^−1^ and a pore size of 1.5 nm were achieved through electropolymerization with concurrent polymer-film deposition (Figure 4). This neutral porous polymer (**PBC**) exhibited extremely low work function-selective electron flow. After ionic ligation of (n-C_4_H_9_)_4_NF and electro-oxidation of carbazole units, the ionic network (**OFPBC**) exhibited a significantly increased work function and turned into hole conduction, which offered an unprecedented example with robust chemical and thermal stability for electrode interlayers in energy-converting solar cells and light-emitting diodes.

Very recently, our group also prepared a new family of Lewis acid triarylborane-based conjugated porous polymers, which were readily converted to the boron-containing anionic conjugated porous polymers via the Lewis acid-base interaction, upon the treatment with tetrabutylammonium fluoride (TBAF). Furthermore, the tetrabutylammonium cation in the network could be replaced by various transition metal cations by ion exchange to form the transition metal-loaded boron-containing conjugated porous polymers. As an example of cobalt(II)-loaded porous polymers, they enabled efficient catalysis of homocoupling reactions of Grignard reagents with good size selectivity, associated with the pore structures of the resulting anionic porous polymers. On the basis of such a strategy, one even could introduce more than one transition metal ion into the as-prepared anionic porous polymer networks, constructing versatile heterogeneous catalytic systems. Along this line, some works are in progress in our group [105].

### 2.3. BODIPY-Based Building Blocks

For the tri-coordinate boron, the empty p orbital on the boron center allows it excellent electron receptivity, which provides the opportunity for the coordination with a heteroatom, e.g., oxygen, nitrogen, to form tetra-coordinate boron-based compounds [106]. Among them, boron dipyrromethene (BODIPY), comprising a four-coordinated boron center chelated by a bidentate heterocyclic ligand and two fluorine atoms, is an important chelated borate dye [107,108]. It exhibits rich photophysical properties, including high absorption coefficients, good fluorescence quantum yields, relatively narrow absorption and emission bands and, thus, always serves as the functional building blocks for the formation of oligomers, polymers and metal organic frameworks (MOFs) [109,110], demanding for organic solar cells, organic light-emitting diodes, sensing and imaging, *etc.* [111]. Encouraged by these successes, BODIPY-based conjugated porous polymers are being explored.

**Figure 5:**
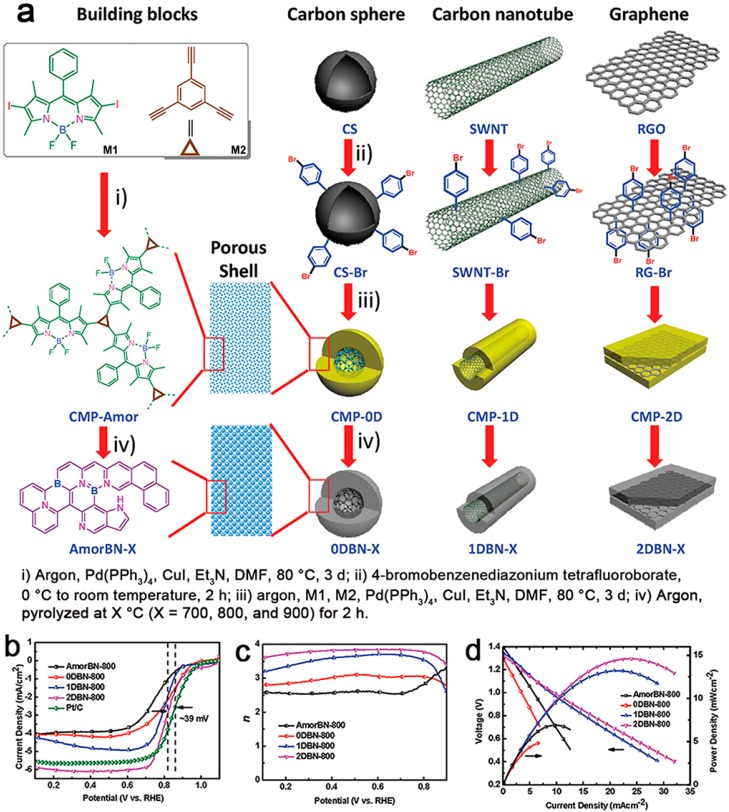
A synthesis illustration of BODIPY-based porous polymers (conjugated microporous polymers (CMP)) **CMP-0D**, **CMP-1D** and **CMP-2D** (**a**); linear sweep voltammetry curves of **AmorBN-800**, **0DBN-800**, **1DBN-800**, **2DBN-800** and Pt/C in O_2_-saturated 0.1 M KOH solution at 1600 rpm at a scan rate of 5 mV·s^−1^ (**b**); electron-transfer number for **AmorBN-800**, **0DBN-800**, **1DBN-800** and **2DBN-800** as a function of the electrode potential (**c**); polarization curve (V–i) and the corresponding power density plot of the battery using **AmorBN-800**, **0DBN-800**, **1DBN-800** and **2DBN-800** as the cathode catalysts (**d**). Reprinted with permission from [46]. Copyright 2015, Wiley-VCH.

In 2015, Zhang and coworkers reported a series of BODIPY-based CMPs (**CMP-0D**, **CMP-1D** and **CMP-2D**) with the controlled dimensionalities, in which bromo-functionalized carbon fullerene (0D), single-walled carbon nanotubes (1D) and reduced graphene oxide (2D) were used as the templates, and 2,6-diiodo-1,3,5,7-tetramethyl-8-phenyl-4,4-difluoroboradiazaindacene and 1,3,5-triethynylbenzene were copolymerized and grafted onto the templates through the Sonogashira–Hagihara coupling reaction (Figure 5) [46]. The resulting **CMP-nD** had well-defined nanosphere, nanotube and nanosheet morphologies with controlled thickness on nanocarbon by adjusting the feed ratios of the template and monomers. After employing 0D, 1D and 2D nanostructured carbon templates, the *S*_BET_ of **CMP-nD** were 622, 614 and 593 m^2^·g^−1^, respectively, which were higher than that of CMP obtained without the use of a template (574 m^2^·g^−1^). The pore structures of **CMP-nD** were not affected by the templates, in comparison with that of pure CMP. After pyrolysis treatment, the resulting boron-containing porous polymers **CMP-nD** were facile to be converted to the B/N co-doped porous carbon materials with controlled dimensionalities, which exhibited promising electrocatalytic performance due to the synergistic effects from boron and nitrogen. We found that the B/N co-doped porous carbons possess high B/N doping contents (N = 5.1 to 8.6 wt %, B = 0.6 to 0.9 wt %) and exhibited efficient catalytic performance for the oxygen reduction reaction (ORR). More importantly, the structure–property relationship between the dimensions of porous carbon and electrochemically-catalyzed ORR was well established in an order of 2D > 1D > 0D. Owing to ORR performance, these porous carbon materials were used as the air electrodes in zinc–air batteries. As an example, the current density and peak power density of a 2D porous carbon-based battery could reach to 23.9 and 14.6 mW·cm^−2^ at 0.61 V, respectively.

**Figure 6:**
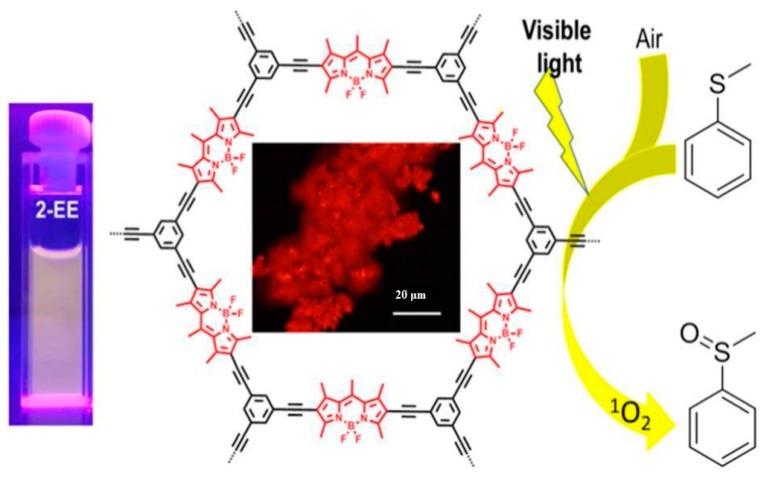
A structural illustration of **CMPBDP** with its fluorescence and catalytic performance. Reprinted with permission from [92]. Copyright 2016, American Chemical Society.

Using a similar synthesis strategy, Liras *et al.* [92] synthesized BODIPY-based conjugated porous polymer (**CMPBDP**) in DMF by using 1,3,5,7,8-pentamethyl-4,4-difluoro-4-bora-3a,4a-diaza-s-indacene and 1,3,5-triethynylbenzene as building blocks in a molar ratio of 1.5: 1 (Figure 6). Although the polymer network was amorphous, the idealized geometry of **CMPBDP** optimized by Materials Studio 6.0 using “universal” as the force field showed a planar configuration, thus leading to the laminar morphology. The *S*_BET_ of **CMPBDP** is 299 m^2^·g^−1^ with a micropore diameter of 1.3 nm and an average mesoporous value of 3.7 nm. Owing to the existence of BODIPY, **CMPBDP** not only showed highly red fluorescence with λ_em_ of 560 nm, even in the solid state, but also could be used as a photocatalyst for selective oxidation of thioanisole. Reaction with 0.1 mol % of **CMPBDP**, the corresponding sulfoxide product was obtained in a yield of 99% after 24 h of reaction, which was higher than those of model compounds. This catalytic performance was comparable with the reported BODIPY analogues, like iodine-functionalized BODIPY, dimeric BODIPY and other photosensitizers of Ru(bpy)_3_Cl_2_. The recycling performance of **CMPBDP** was also evaluated, and the activity and selectivity of **CMPBDP** could be maintained within 50 h of irradiation.

### 2.4. Tetraphenylborate-Based Building Blocks

Tetraphenylborate is an important weakly-coordinating anionic borate, which can be used for stabilizing the electrophilic cation species to achieve their catalytic activity in the carbon-carbon formation reaction [112,113]. Wuest and coworkers reported the anionic porous supramolecular networks from tetraphenylborates bearing multiple hydrogen-bonding sites [96]. This anionic network was crystalline, in which 26% of the volume of the crystals was occupied by the ordered tetraphenylborates, while 74% was available for including cations or guests. Interestingly, the resulting channels were aligned with the c axis and had triangular cross-sections measuring approximately 8 × 12 Å^2^ at the narrowest points. Thus, the porosity of the anionic network was greater than those of the networks built from the neutral analogues.

**Figure 7:**
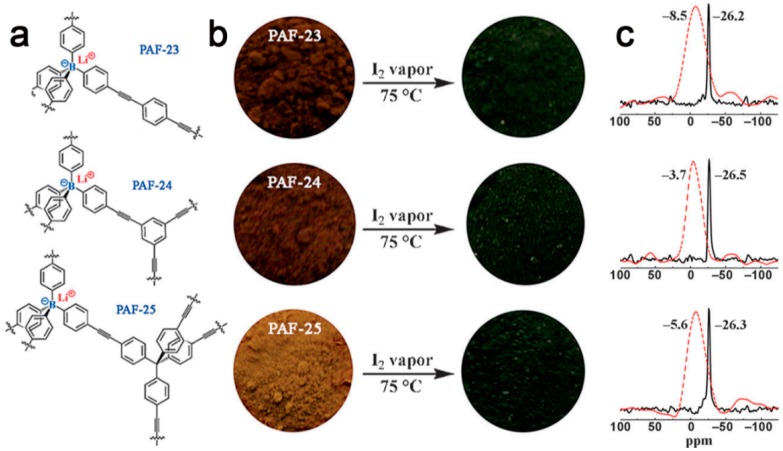
A schematic illustration of triphenyl borate-based porous polymers **PAF-23**, **PAF-24**, and **PAF-25** (**a**); photographs of **PAF-23**, **PAF-24** and **PAF-25** when adsorbing iodine (**b**); the change of solid-state ^11^B NMR spectra of **PAF-23**, **PAF-24** and **PAF-25** after adsorbing iodine (**c**). Reprinted with permission from [94]. Copyright 2015, Wiley-VCH.

In 2015, Zhu’s group [94] presented the design and synthesis of a series of charged porous polymers (**PAF-23**, **PAF-24**, and **PAF-25**) comprising the charged tetrahedral lithium tetrakis(4-iodophenyl)borate with different alkyne monomers, like 1,3,5-triethynylbenzene, 1,4,-diethynylbenzene and tetrakis(4-ethynylphenyl)methane (Figure 7). Owing to the efficient Sonogashira–Hagihara coupling polymerization, these porous polymers showed high polymerization degrees and amorphous architectures. The values of their *S*_BET_ were 82, 136 and 262 m^2^·g^−1^, respectively, with pore sizes of 0.44 to 0.47 nm, calculated by the Horvath–Kawazoe (HK) method. After being exposed to the iodine vapor, the color of the powders became progressively darker; correspondingly, the boron signals were significantly shifted to the low field (from −26 ppm to −5 ppm) and became broader after iodine capture. These results indicate that the central boron atoms in networks show high affinity with iodine molecules. The I_2_ uptakes of these charged porous polymers were evaluated in a pre-weighed glass vial at 75 °C and ambient pressure. The results showed that per gram of **PAF-23**, **PAF-24** and **PAF-25** could adsorb approximately 2.71, 2.76 and 2.60 g of iodine, respectively, which was the highest sorption values compared to those of the reported materials. The reason might be attributed to the effective sorption sites, including an ionic bond, phenyl ring and triple bond, for the high affinity of iodine. These materials were recyclable and could be reused for iodine capture.

**Figure 8:**
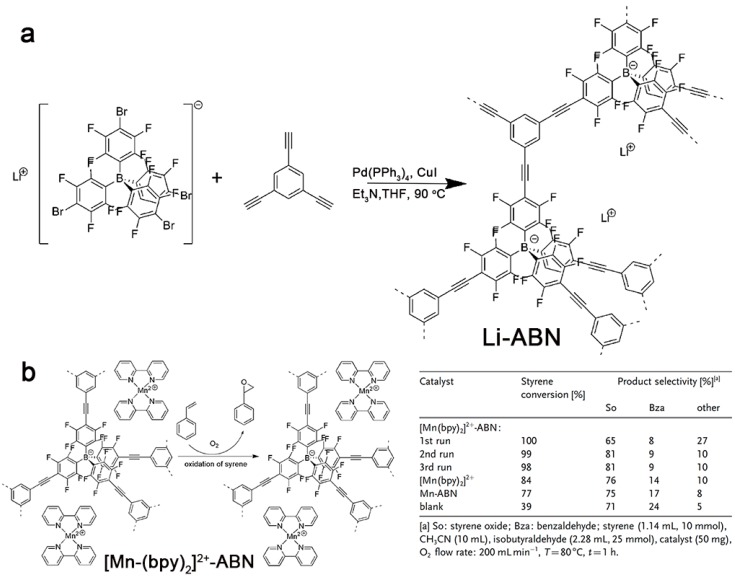
A synthesis route of **Li-ABN** (**a**); structural illustration of **[Mn-(bpy)_2_]^2+^-ABN** and its catalysis performance of styrene oxidation (**b**). Reprinted with permission from [93]. Copyright 2013, Wiley-VCH.

Compared to non-fluorinated tetraphenylborate anion, tetrakis(pentafluorophenyl)borate shows high chemical and photochemical stability, which has been widely applied as a building block in linear polymers or dendrimers [114,115]. Thomas *et al.* failed to synthesize homogeneous porous polymers via a Yamamoto coupling polymerization of lithium tetrakis(4-bromo-2,3,5,6-tetrafluorophenyl)borate (Li[B(C_6_F_4_Br)_4_]) [93]. Therefore, they prepared the conjugated porous copolymer (**Li-ABN**) containing Li[B(C_6_F_4_Br)_4_] with 1,3,5-triethynylbenzene by Sonogashira–Hagihara coupling polymerization (Figure 8). This network showed a high *S*_BET_ of 890 m^2^·g^−1^ and a pore volume of 0.61 cm^3^·g^−1^, which was higher than that of uncharged analogues. Using the ”ship-in-a-bottle” strategy, Li cation in **Li-ABN** could be exchanged by other cations. As an example of manganese(II) bipyridine complexes ([Mn-(bpy)_2_]^2+^) with good catalytic properties, the obtained **[Mn-(bpy)_2_]^2+^-ABN** could be used as catalysts for the oxidation of alkenes and alkanes. Within one hour, the complete conversion of styrene was observed, and the selectivity of the product could reach to 81%, even at the third run. These results demonstrated that the catalytic performance of **[Mn-(bpy)_2_]^2+^-ABN** was better than other catalysts. In addition, the low solubility of **[Mn-(bpy)_2_]^2+^-ABN** in acetonitrile led to its recyclability and stability during the first three runs.

Long and coworkers [95] reported the anionic tetrakis(phenyl)borate conjugated porous polymers by Sonogashira cross-coupling of tetrakis(4-iodophenyl)borate, tetrakis(4-iodo-2,3,5,6-tetrafluorophenyl)borate or tetrakis(4-bromo-2,3,5,6-tetrafluorophenyl)borate with 1,4-diethynylbeznene and its tri(ethylene glycol)-substituted derivative. The good conductivity (2.7 × 10^−4^ S·cm^−1^), mild activation energies (0.28 eV) and strong ion-conducting transport (*t*_Li+_ = 0.93) had been obtained for these anionic porous polymers.

## 3. Networks with Different Boron-Based Linkers

### 3.1. B-O Type Linkers

Owing to its low toxicity to transition metal catalysts, boronate groups have been widely used as the functional groups in carbon-carbon cross-coupling reactions, e.g., Suzuki reaction. The formation of boronate groups also can be applied for the synthesis of porous polymers with high crystallinity, for example COFs, due to the dynamic covalent bond of boronate linkage. As a benchmark work, Yaghi and co-workers reported the first type of COFs (**COF-1**) [10], which was synthesized by self-condensation reactions of phenyl diboronic acid with planar six-membered B_3_O_3_ rings. As a result of the reversible covalent bond of boroxine from the dehydration of boronic acid, this COF showed high crystallinity with the expanded porous 2D graphitic layers, and a Brunauer-Emmett-Teller (BET) surface area of 711 m^2^·g^−1^ and an average pore size of 0.7 nm were obtained. Besides boroxine, boronate esters and borosilicate also could be used for the construction of boronate-based COFs [10,11]. For example, 2D **COF-5** containing boronate ester linkages was synthesized via the co-condensation of diboronic acid and hexahydroxy triphenylene with a longer conjugated length, leading to the higher S_BET_ of 1590 m^2^·g^−1^ with the comparison of **COF-1**. The higher surface area of 4210 m^2^·g^−1^ was obtained for 3D boroxine-based COF constructed from self-condensation of diboronic acid with tetra(4-dihydroxyborylphenyl)silane.

**Figure 9:**
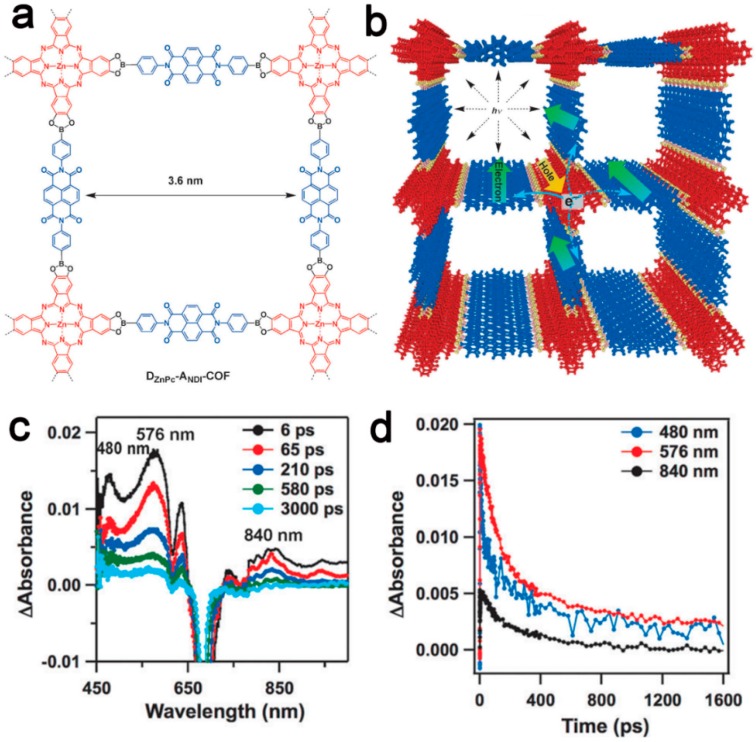
A structural illustration of the donor–acceptor covalent organic framework (COF) (**D_ZnPc_-A_NDI_ COF**) (**a**); slipped stacking of **D_ZnPc_-A_NDI_ COF** and the illustration of its electron transport (**b**); femtosecond transient absorption spectra of **D_ZnPc_-A_NDI_ COF** at different time intervals in DMF (**c**); time profiles of the transient bands at 480, 576 and 840 nm (**d**). Reprinted with permission from [121]. Copyright 2016, Wiley-VCH.

To date, great success in constructing boronate-based COFs by various synthetic strategies and building blocks was achieved [47,116]. With tunable chemical and physical properties, these COF materials, even with the non-conjugated boronate linkers, were exploited as promising candidates for applications in optoelectronics, including luminescent materials and organic semiconductors. In organic semiconductors, the charge transfer is not only along the delocalized backbone of conjugated polymers, but also goes through the columnar molecular arrangements by using a thermally activated hopping mode [117]. Jiang and coworkers synthesized pyrene-functionalized COFs (**PPy-COF** and **TP-COF**) via self-condensation of pyrene-2,7-diboronic acid (PDBA) and co-condensation PDBA with 2,3,6,7,10,11-hexahydroxytriphenylene [118,119]. Both **PPy-COF** and **TP-COF** possess highly ordered 2D eclipsed structures, which enable forming a conductive path for the relatively high electric current. Using a porphyrin as the building block, the possibilities of carrier transportation of COFs could be adjusted from hole conduction to ambipolar and electron conduction by coordination of metal (copper and zinc) with porphyrin, owing to the different electron transport abilities of the metal channels in H_2_-COF, CuP-COF and ZnP-COF [120]. Recently, Jiang *et al.* [121] reported donor–acceptor COF consisting of boronate ester linkers, zinc phthalocyanines as electron donors and naphthalene diimides as acceptors (Figure 9). This **D_ZnPc_-A_NDI_ COF** provided self-sorted, bicontinuous columnar arrays and constitutes a periodically-structured heterojunction system, in which each donor column was interfaced with four acceptor columns. Therefore, charge separation and exciton transfer would be realized when the exciton was generated directly by light absorption, accounting for the long-distance charge delocalization and exceptional long-term charge retention in the aligned bicontinuous π-columns, which provided them as promising high performance semiconducting materials for the application in photo-induced energy conversion.

**Figure 10:**
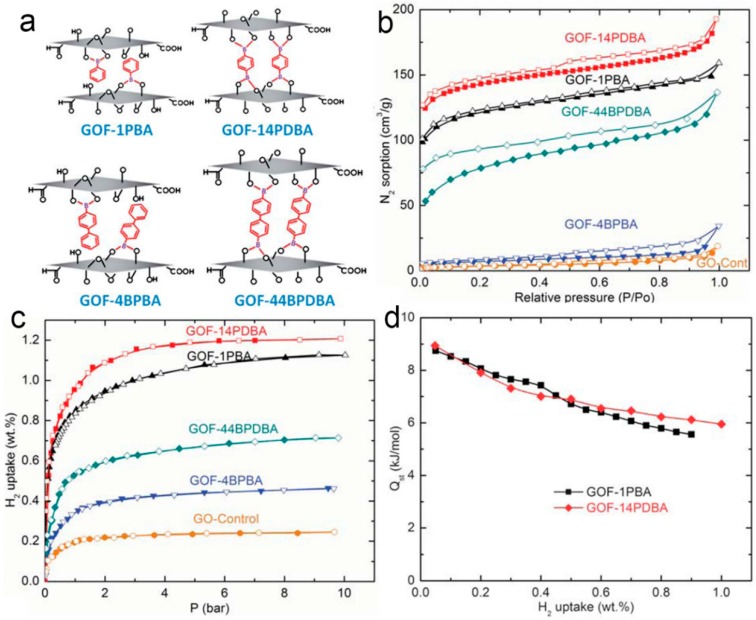
A structural illustration of graphene oxide frameworks (GOFs) with various types of phenylboronate pillaring units (**a**); nitrogen adsorption (filled) and desorption (empty) isotherms of GOFs measured at 77 K (**b**); hydrogen adsorption isotherms of GOFs at 77 K (**c**); isosteric heat of **GOF-1PBA** and **GOF-14PDBA** for capturing H_2_ (**d**). Reprinted with permission from [122]. Copyright 2011, The Royal Society of Chemistry.

Using graphene oxide as a building block, Srinivas *et al.* [122] synthesized a range of porous graphene oxide frameworks (GOFs) by cross-linking of oxygen functional groups on the GO layers with various boronic acids to form the linear boronate ester linkers in a solvothermal reaction (Figure 10). The GOFs showed periodic layered structures with largely expanded interlayer spacing. Owing to the existence of strong boronate-ester bonds between the GO layers, the thermal stability of GOFs is higher than that of precursor GO. This frameworks exhibited a reasonably higher surface area of about 470 m^2^·g^−1^ compared to the *ca*. 10 m^2^·g^−1^ for GO. The H_2_ uptake of all of the GOFs at 77 K was evaluated, and the obtained H_2_ uptake capacities (1.2 to 0.4 wt %) of GOFs are relatively high in comparison with other porous materials with respect to the similar BET surface area.

### 3.2. B-N-Type Linkers

**Figure 11:**
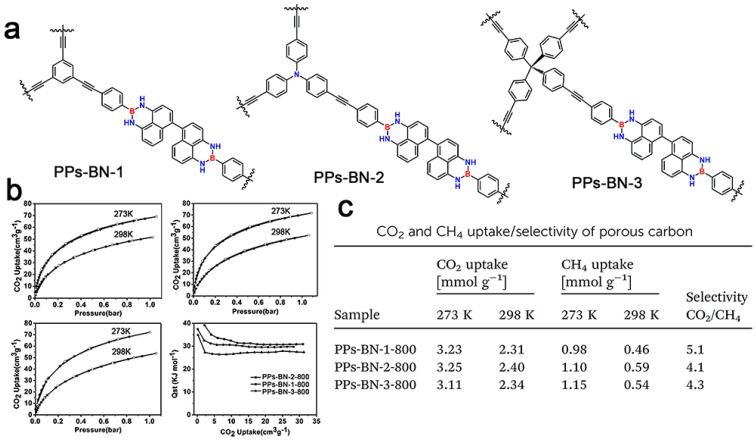
A structural illustration of **PPs-BN-1**, **PPs-BN-2** and **PPs-BN-3** (**a**); CO_2_ adsorption isotherms and isosteric heat of **PPs-BN-1-800**, **PPs-BN-2-800** and **PPs-BN-3-800** at 273 K and at 298 K (**b**); CO_2_ and CH_4_ uptake/selectivity of porous carbon (**c**). Reprinted with permission from [85]. Copyright 2015, The Royal Society of Chemistry.

In comparison with the boronate ester linker, six-membered heterocyclic 1,3,2-diazaborine composed from boronic acid with diamino compounds is much more stable both in moist air and organic solvents. However, as far as we know, there are very few BN-containing conjugated porous polymers documented so far. In 2015, Zhang *et al.* pre-prepared 1,3,2-diazaborine-containing building block via condensation reaction of 4,4,5,5-tetraamino-1,10-binaphthyl with 4-bromo-phenylboronic acid in a yield of 73% [85]. This key monomer was further used to synthesize B, N-containing porous conjugated polymers (**PPs-BN-*i***) by Sonogashira–Hagihara cross-coupling copolymerization with 1,3,5-triethynylbenzene, tris(p-ethynylphenyl) amine and tetrakis(4-ethynylphenyl)methane, respectively (Figure 11). These porous polymers showed low porosities of 16, 32 and 51 m^2^·g^−1^ for **PPs-BN-1**, **PPs-BN-2** and **PPs-BN-3**, respectively, which was caused by the low rigidity of the naphthyl moieties in polymeric networks. After pyrolysis at 800 °C under a nitrogen atmosphere for 2 h, carbon materials deriving from **PPs-BN-*i*** exhibited a high degree of graphitization with increasing BET surface areas of 215, 291 and 268 m^2^·g^−1^, respectively. The obtained B/N co-doped porous carbons showed excellent CO_2_ adsorption, and the CO_2_ uptakes of **PPs-BN-1-800**, **PPs-BN-2-800** and **PPs-BN-3-800** reached to 3.23, 3.25 and 3.11 mmol·g^−1^, respectively, at 273 K and 1 bar pressure. The B/N co-doped porous carbon **PPs-BN-3-800** with a low surface area of 268 m^2^·g^−1^ offered a higher CO_2_ uptake than N-doped porous carbon FCDTPA-700 (BET = 417 m^2^·g^−1^) under the same conditions, suggesting that the increased polarization arising from the B/N co-doped effect might enhance the basicity of the backbone of the resulting materials, which was likely beneficial to CO_2_ capture. In addition, the selective gas absorption of B/N co-doped porous carbons was also evaluated. Due to the different polarity between methane and carbon dioxide, the highest selectivity of CO_2_/CH_4_ was achieved for **PPs-BN-1-800** in a ratio of 5.1/1 at 298 K and 1 bar, which was comparable to those of the reported porous materials.

Kahveci *et al.* had designed and synthesized a kind of five-membered diazaborole-linked porous organic polymer (**DBLP**) by utilizing the condensation reaction of diamines and boronic acids [123]. These microporous **DBLPs** exhibited high surface areas in the range of 730 to 1120 m^2^·g^−1^. **DBLPs** can store CO_2_ up to 4.48 mmol·g^−1^ (at 273 K and 1 bar) with high (35 kJ·mol^−1^) isosteric heats of adsorption. This high uptake and binding affinity for CO_2_ might arise from the rich nitrogen content and the microporous nature of the polymers. Other novel properties, including high H_2_ uptake 2.13 wt % at 77 K and 1 bar and moderate CO_2_/N_2_ selectivity were obtained.

## 4. Conclusions and Perspectives

In this feature article, we have witnessed the significant progress in the synthesis and application of boron-containing conjugated porous polymers in recent years. The structures of these porous polymers could be tailorable by changing the boron-based building blocks (like triarylborane derivatives, BODIPY derivatives and triarylborate derivatives), the linkage modes (e.g., carbon-carbon linkage and boronate linkage) and polymerization reaction conditions. With respect to the full-carbon analogues, incorporation of boron atoms into the skeletons of the conjugated porous polymers can take unique advantage of their empty p_π_ orbitals to offer Lewis acidity, extended π-conjugated systems and an electron-deficient character. As a consequence, the resulting boron-containing conjugated polymers are rendered with very rich physical properties, including solvatochromic behavior, bright fluorescent properties in the solid state, specific recognition of anions, strong dipole–quadrupole interaction with CO_2_, ion exchange capability and a narrow energy gap, thus making them widely applicable for luminescent organic devices, selective sensors, gas storage and separation, catalysts, energy storage and conversion.

To date, a great deal of accomplishments and progress have already been achieved in the area of boron-containing conjugated porous polymers. However, some primary challenges still exist as follows: (1) the design and synthesis of novel boron-based building blocks; (2) the exploration of new boron-based linkages governed by dynamic covalent bonding; (3) the improvement of the stability of boron-based porous polymers against moisture and air; (4) the establishment of the boron-based multi-stimuli-responsive systems. Provided that these difficulties are overcome, boron-based conjugated porous polymers serve as promising candidates in a much larger scope.
